# Inflammatory breast cancer among advanced breast malignancies: diagnostic, microenvironmental, and therapeutic challenges of the rare amongst the common

**DOI:** 10.3389/fonc.2026.1935087

**Published:** 2026-10-01

**Authors:** Shayla Murray, Matthew Becker, Bora Lim

**Affiliations:** 1 Department of Medicine, Drexel University College of Medicine, Philadelphia, PA, United States; 2 Department of Biomedical Sciences, University of South Carolina School of Medicine Greenville, Greenville, SC, United States; 3 Department of Breast Medical Oncology, The University of Texas MD Anderson Cancer Center, Houston, TX, United States

**Keywords:** immunotherapy, inflammatory breast cancer, metastatic breast cancer, triple-negative breast cancer, tumor microenvironment

## Abstract

Breast cancer is one of the most commonly diagnosed malignancies among women worldwide and is frequently curable when identified at an early stage. However, advanced and metastatic disease continue to account for a substantial proportion of breast cancer-related deaths, particularly among patients with aggressive subtypes such as inflammatory breast cancer (IBC), a clinicopathologic entity that spans hormone receptor-positive, HER2-positive, and triple-negative disease. Although advances in molecular profiling and systemic therapies have improved outcomes, substantial challenges persist in identifying patients at risk for recurrence, accurately assessing treatment response, and determining optimal therapeutic strategies. These challenges are driven by marked tumor heterogeneity and the dynamic interplay between malignant cells and their surrounding tumor microenvironment (TME). This review examines key barriers to the diagnosis and management of IBC, set against the broader landscape of advanced breast cancer. It discusses the clinical significance of late metastatic relapse, rapidly progressive disease, shortcomings of current prognostic frameworks, and the difficulties associated with monitoring metastatic involvement. Also highlighted are emerging approaches, including circulating tumor DNA (ctDNA) and other liquid biopsy technologies, that may enhance risk stratification, longitudinal surveillance, and detection of molecular resistance; these applications remain investigational, and established clinical approaches are distinguished from emerging strategies throughout. The TME is further explored as a critical determinant of tumor behavior, with emphasis on lymphoid and myeloid immune cells, stromal components, and immune checkpoint pathways that promote immune evasion and therapeutic resistance. Finally, this review surveys evolving treatment paradigms, including immunotherapeutic approaches, RNA-based diagnostics and therapeutics, most of which remain preclinical, and antibody-drug conjugates, which are expanding the therapeutic landscape for advanced disease. Together, these developments underscore the potential of integrating molecular, immunologic, and circulating biomarker data to enable more precise disease characterization and treatment selection, as essential for developing more effective, personalized strategies for patients with IBC and other advanced breast cancers.

## Introduction

Breast cancer remains one of the most prevalent malignancies worldwide, affecting predominantly women but also occurring in men. While approximately 75-80% of breast cancers are curable when detected early, outcomes for advanced breast cancer remain poor, largely due to tumor heterogeneity and the dynamic nature of disease progression (1, 2). Among these, inflammatory breast cancer (IBC) represents one of the most aggressive and biologically distinct subtypes, characterized by rapid progression, early metastasis, and limited responsiveness to conventional therapies. IBC is defined clinicopathologically rather than molecularly: it spans hormone receptor-positive, HER2-positive, and triple-negative disease, and although it is enriched for aggressive receptor subtypes, IBC and triple-negative breast cancer (TNBC) are distinct, partially overlapping categories that should not be treated as interchangeable (3, 4). Notably, effective targeted treatment options for metastatic IBC remain scarce.

A growing body of evidence suggests that interactions between tumor cells, the tumor microenvironment (TME), and the host immune system play a central role in driving disease progression, therapeutic resistance, and clinical outcomes. However, the underlying molecular and cellular mechanisms governing these interactions are not yet fully understood, particularly in aggressive subtypes such as IBC and TNBC (3, 4). In parallel, current diagnostic and prognostic tools remain insufficient for accurately predicting recurrence, monitoring disease evolution, or guiding personalized treatment strategies, a critical unmet clinical need (4–6).

This review focuses on IBC, using the broader advanced breast cancer landscape, in particular the late-relapsing behavior of hormone receptor (HR)-positive disease and the infiltrative biology of invasive lobular carcinoma, as the comparative backdrop against which the compressed kinetics of IBC are defined. The choice of focus is deliberate: IBC is among the most lethal presentations of primary breast cancer, is rare enough to be underrepresented in the trials that define standard care, and has no approved therapy specific to the disease, a combination that concentrates the central challenges of advanced breast cancer, early recognition, accurate monitoring, and rational treatment selection, into a single entity. Specifically, it explores limitations of existing diagnostic and monitoring approaches, examines the roles of the TME and immune components in disease progression, and evaluates emerging molecular and immunotherapeutic strategies. It differs from existing reviews of IBC biology, liquid biopsy, and the breast TME in connecting three literatures usually treated separately: it frames disease kinetics as defining a molecular interception window whose width is subtype-dependent, positions the IBC TME as the biological explanation for why that window is narrow, and ties both to therapy selection. Throughout, established clinical approaches are explicitly distinguished from investigational strategies.

## Literature search and selection

This article is a narrative mini-review. Literature was identified through searches of PubMed and Embase, supplemented by ClinicalTrials.gov for trial status, covering January 2000 through May 2026, with emphasis on work published in the last five years. Principal search terms included “inflammatory breast cancer,” “metastatic breast cancer,” “circulating tumor DNA,” “minimal residual disease,” “liquid biopsy,” “tumor microenvironment,” “tumor-infiltrating lymphocytes,” “tumor-associated macrophages,” “tumor-associated neutrophils,” “antibody-drug conjugate,” and “microRNA,” alone and in combination. Only English-language publications were considered. Study types included randomized and non-randomized clinical trials, prospective and retrospective cohort studies, meta-analyses, preclinical investigations, and reviews; conference abstracts and clinical trial registry entries were included only where peer-reviewed publications were unavailable and are identified as such in the text. Selection was narrative rather than systematic, prioritizing recent primary studies, prospective trials, and contemporary guidelines, and reflects the authors’ judgment of relevance to the review’s scope. The date of the last search was May 7, 2026.

## Diagnostic and monitoring approaches

### Delayed recurrence and misleading prognosis

Metastatic recurrence of breast cancer can present in clinically unexpected ways, including isolated involvement of lymph node basins, underscoring the inherent unpredictability of disease progression and the challenges in long-term management (1). Patients initially diagnosed with early-stage or *in situ* disease, often counseled as having an extremely low risk of recurrence, may ultimately develop distant metastatic disease. Such cases highlight a critical limitation in current prognostic frameworks, which may underestimate long-term risk despite favorable initial presentation and adherence to recommended surveillance and lifestyle measures.

A particularly striking observation in breast cancer epidemiology is the substantial contribution of early-stage disease to the population of patients who eventually develop metastatic recurrence. A significant proportion of patients presenting with stage IV disease were initially diagnosed with stage I or II tumors, most commonly hormone receptor (HR)-positive subtypes (7). In the EBCTCG meta-analysis of over 60,000 women with ER-positive disease, distant recurrence continued to accrue steadily throughout years 5 to 20 after diagnosis, with 20-year risks of 13% to 41% depending on initial stage and grade, even after five years of endocrine therapy (7). This phenomenon reflects the complex and often deceptive biology of HR-positive tumors, which are characterized by the capacity for prolonged dormancy followed by late relapse, sometimes occurring decades after completion of adjuvant therapy. Despite appropriate initial treatment, including endocrine therapy, a subset of these tumors acquires resistance over time and ultimately progresses to metastatic disease, exposing fundamental limitations in current therapeutic and prognostic strategies.

Current clinical risk stratification models, largely based on tumor stage, grade, and receptor status, remain insufficient to accurately identify patients at risk for late recurrence (8). Notably, most existing tools emphasize recurrence risk within the first 5–9 years following diagnosis, thereby failing to account for the persistent long-term risk observed in HR-positive disease (9). This gap underscores the need for more dynamic and biologically informed approaches to risk assessment. Emerging strategies, including molecular and genomic profiling, as well as longitudinal monitoring using circulating tumor DNA (ctDNA) and other markers of minimal residual disease (MRD), offer the potential to detect subclinical relapse earlier and refine individualized surveillance (10, 11). In high-risk HR-positive disease, ctDNA can precede clinical recurrence by a median of many months to more than a year (12), and in stage III IBC, molecular residual disease has been reported to precede radiographic confirmation of recurrence, although this observation derives from a conference abstract and awaits full publication (10).

Whether detection should trigger intervention is a separate question, and the distinction matters: ctDNA-based MRD assays have demonstrated analytical validity and prognostic (clinical) validity in multiple settings, but clinical utility, meaning improved outcomes when treatment decisions are changed on the basis of the assay, is only beginning to be established. The strongest evidence to date comes from ctDNA-guided switching rather than escalation: in SERENA-6, switching the endocrine partner upon emergence of ESR1 mutation in ctDNA, before radiographic progression, improved progression-free survival in HR-positive metastatic disease (13). By contrast, in c-TRAK TN, a ctDNA surveillance strategy in early-stage triple-negative disease, a majority of patients with a positive ctDNA result already had overt metastatic disease on staging, illustrating how quickly the interception window can close in aggressive subtypes (14). Earlier introduction of CDK4/6 inhibition or other escalation at molecular relapse therefore remains a hypothesis under prospective evaluation, and should be pursued within clinical trials rather than adopted into routine practice.

### Rapid progression and unique challenges

While late recurrence remains a well-recognized clinical challenge, an equally significant but less emphasized phenomenon is the rapid development of metastatic disease during initial treatment. This pattern of early progression undermines therapeutic efficacy from the outset and reflects a biologically distinct and highly aggressive disease course. In contrast to the relatively indolent behavior of late-relapsing hormone receptor-positive tumors, these rapidly progressing cancers often exhibit intrinsic resistance to standard therapies and quickly become refractory to conventional treatment regimens.

This aggressive trajectory is most associated with high-risk histologic subtypes, including inflammatory breast cancer (IBC), metaplastic breast cancer (MpBC), and neuroendocrine breast carcinoma (NEBC), all of which are characterized by unique molecular drivers and distinct clinical behavior (15). However, early progression is not confined to these rare subtypes. Notably, similar patterns can also be observed in more common entities such as invasive lobular carcinoma (ILC), which is traditionally considered more indolent. The diffuse, infiltrative growth pattern of ILC, often driven by loss of E-cadherin, may facilitate early occult dissemination, challenging conventional assumptions regarding disease kinetics and risk stratification (16).

These observations highlight important limitations of standardized, guideline-based treatment approaches, which may not adequately account for aggressive or rapidly evolving disease biology. In this context, a more proactive and individualized strategy is warranted, one that incorporates early genomic profiling, advanced imaging modalities, and multidisciplinary evaluation to better characterize tumor behavior at diagnosis.

### Limitations in precision oncology and therapy optimization

The evolution of precision oncology has transformed the management of breast cancer by enabling treatment strategies tailored to individual tumor biology, genetic profiles, and patient-specific factors. However, the clinical application of precision medicine remains constrained by significant limitations. Responses to targeted and systemic therapies are often heterogeneous and unpredictable, reflecting the complex and dynamic nature of tumor evolution (17). In practice, many patients experience a trial-and-error treatment course, characterized by variable efficacy across therapies and limited responsiveness to chemotherapy or targeted agents.

Beyond clinical outcomes, this iterative treatment process imposes a substantial physical and psychological burden on patients. The cumulative effects of multiple therapies, treatment-related toxicities, and uncertainty surrounding disease trajectory contribute to significant emotional distress and diminished quality of life (18). These challenges are particularly pronounced in patients with metastatic breast cancer, where disease control is often prolonged but rarely curative.

Collectively, these limitations highlight the urgent need for more robust diagnostic approaches and more effective, biologically informed therapeutic strategies. Advances in molecular profiling, real-time disease monitoring (e.g., circulating tumor DNA), and biomarker-driven treatment selection hold promise in reducing therapeutic uncertainty and enabling earlier, more precise intervention (19).

### Monitoring of disease

Bone metastases represent one of the most common and clinically significant sites of disease spread in advanced breast cancer, contributing substantially to morbidity and decreased quality of life (20). As such, accurate detection and monitoring of bone involvement are critical components of metastatic disease management. However, evaluating bone metastases remains particularly challenging due to their heterogeneous biology, often presenting mixed osteolytic and osteoblastic features that complicate both detection and response assessment (21).

A variety of imaging modalities are employed to assess bone metastases, including computed tomography (CT), positron emission tomography/computed tomography (PET/CT), bone scintigraphy, and magnetic resonance imaging (MRI) (21). On conventional imaging, lesions may appear sclerotic, lytic, or mixed, and no single modality is sufficient in all clinical scenarios. As a result, these techniques are frequently used in combination to improve diagnostic accuracy.

In clinical practice, one major challenge is the phenomenon of treatment-induced sclerosis, which may reflect healing rather than disease progression, yet can be radiographically indistinguishable from worsening metastases (22). Additionally, widely used response criteria such as Response Evaluation Criteria in Solid Tumors (RECIST) have limited applicability in bone-predominant disease, as they do not reliably capture changes in non-measurable or sclerotic lesions.

### Liquid biopsies and circulating biomarkers

Serum-based biomarkers have long been utilized in the monitoring of metastatic breast cancer, although their clinical utility remains limited by variability and lack of specificity. Commonly measured tumor markers, including carcinoembryonic antigen (CEA) and cancer antigen 15-3 (CA15-3), may provide adjunctive information regarding disease burden and treatment response; however, fluctuations in these markers are not always reliable indicators of progression or therapeutic efficacy (23). In parallel, laboratory parameters such as calcium, phosphorus, and magnesium are routinely monitored in patients receiving bone-targeted therapies, including bisphosphonates and RANK-ligand inhibitors, both to assess treatment-related effects and to detect complications of bone involvement (24). Renal function markers (BUN and creatinine), as well as enzymes such as lactate dehydrogenase (LDH) and alkaline phosphatase (ALP), may also be followed, though they lack specificity for disease monitoring (25).

More recently, liquid biopsy approaches, including the analysis of circulating tumor DNA (ctDNA) and circulating tumor cells (CTCs), have emerged as promising tools for real-time, noninvasive assessment of tumor dynamics (26, 27). Unlike traditional serum markers, ctDNA and CTCs offer the potential to capture tumor-specific molecular alterations and provide earlier indications of treatment response or emerging resistance (27). These markers may be particularly valuable in the context of bone-dominant disease, where conventional imaging and response criteria are limited. Integration of liquid biopsy data with radiographic findings may enhance the accuracy of disease monitoring and allow for more timely therapeutic adjustments (27). However, the incorporation of these technologies into routine clinical practice remains an area of ongoing investigation, with challenges related to standardization, sensitivity, and clinical interpretation.

Several limitations deserve explicit statement. False-negative results occur, and ctDNA shedding varies by disease site and histology: bone-dominant disease and lobular carcinoma can shed little ctDNA, precisely the settings in which imaging is also least reliable. Clonal hematopoiesis of indeterminate potential can produce false-positive variant calls unless assays incorporate matched leukocyte sequencing. Tumor-informed assays achieve greater sensitivity than tumor-naive panels but require tissue, bespoke design, and longer turnaround, a meaningful constraint in rapidly progressing disease such as IBC. Inter-assay variability is substantial, and no standardized variant allele frequency or MRD threshold for clinical intervention has been established (14, 27). These caveats do not diminish the promise of liquid biopsy; they define the validation agenda it must still complete.

As illustrated in **Figure 1**, ctDNA and MRD assays cross their detection threshold before disease becomes radiographically apparent, opening a molecular interception window during which the tumor is detectable at the molecular level yet remains occult on conventional imaging. The width of this window depends on disease trajectory: in HR+ disease characterized by dormancy and late relapse, it may extend for months, with ctDNA leading imaging, whereas in inflammatory and other aggressive subtypes, rapid progression and intrinsic resistance narrow the window and can outpace scheduled imaging, as observed empirically in the c-TRAK TN surveillance study (14).

**Figure 1:**
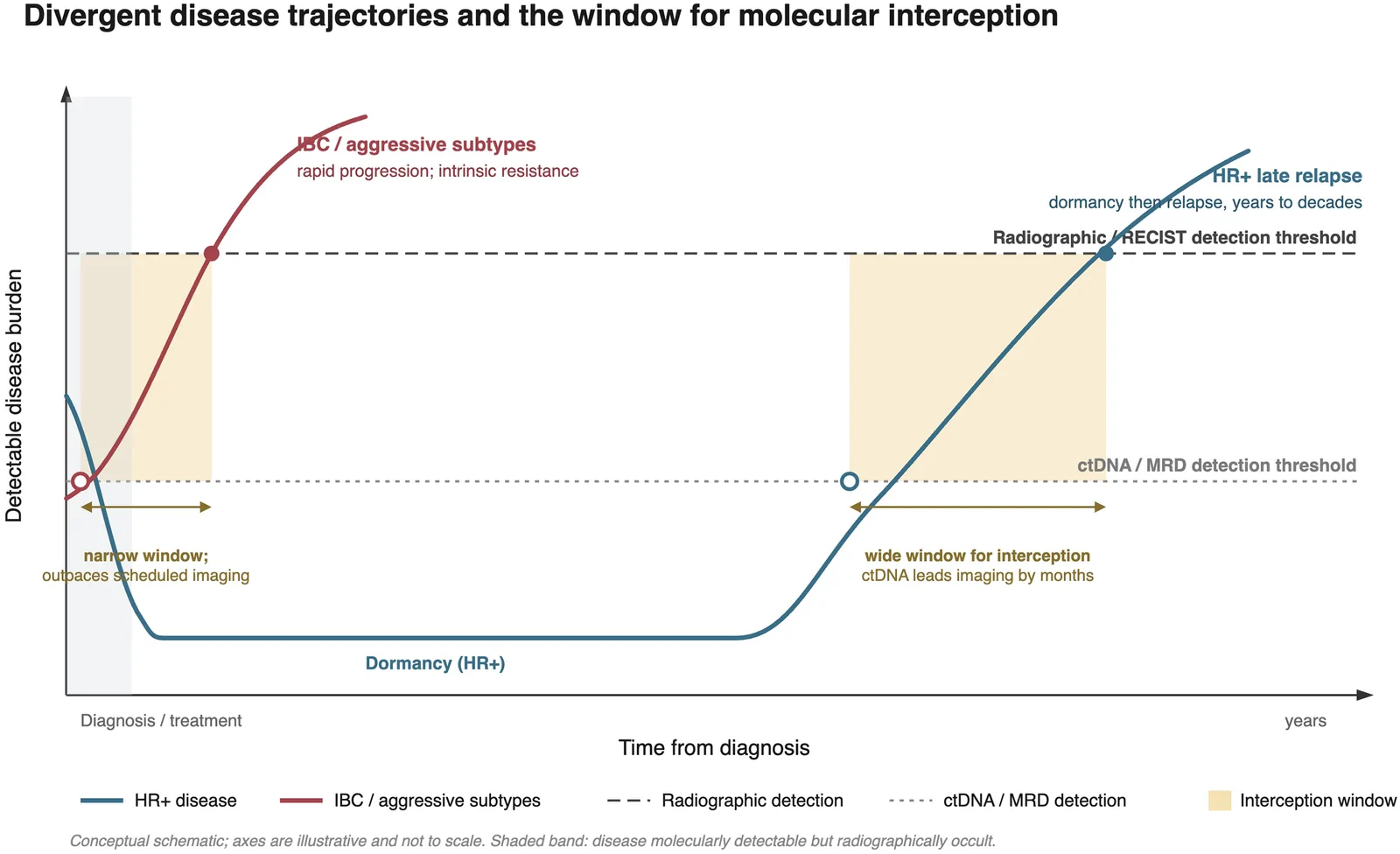
Conceptual model of the molecular interception window across breast cancer trajectories. This schematic is a conceptual, non-quantitative model; curves, thresholds, and time spans are illustrative and do not represent measured clinical values. The upper curve depicts the rapid growth trajectory typical of IBC and other aggressive subtypes; the lower curve depicts the dormancy and late-relapse trajectory typical of HR-positive disease. The horizontal dashed lines indicate, from bottom to top, the detection threshold of ctDNA/MRD assays and the threshold of radiographic detectability; the shaded region between the point at which each curve crosses the molecular threshold and the point at which it crosses the radiographic threshold represents the molecular interception window, whose existence is supported by studies in which ctDNA anticipated clinical or radiographic recurrence (10–12) and whose narrowing in aggressive disease is supported by the c-TRAK TN experience (14). ctDNA, circulating tumor DNA; MRD, minimal residual disease; HR+, hormone receptor-positive; IBC, inflammatory breast cancer. The figure was created using Powerpoint.

## Tumor microenvironment

### Challenges of TME diagnosis and targeting

When detection defines when we recognize aggressive disease, the study of the tumor microenvironment (TME) is a critical starting point for explaining the unique aggressiveness of such disease. However, the TME is a complex network of stromal and immune cells, along with intricate signaling pathways that produce cytokines and other molecules. The TME has garnered significant interest in research due to its critical role in modulating the delicate balance between immune activation and immune tolerance (28, 29). Especially in IBC, the disruption of this balance can contribute to the mechanisms underlying growth and progression; therefore, it has become a key area of research (4). Understanding the recent updates regarding the TME in IBC is crucial for unraveling the complexities of this aggressive form of breast cancer and may provide insights into potential therapeutic strategies. In the sections that follow, the source of each finding, patient cohorts, *in vitro* experiments, animal models, or computational immune deconvolution, is stated, because these evidence types support different strengths of inference; observed associations between immune features and survival are reported as associations, not as demonstrated mechanisms or evidence of therapeutic responsiveness.

### T cells in aggressive breast cancers

Cytotoxic (CD8+) and helper (CD4+) T-lymphocytes are important components of adaptive immunity against cancer. Activated CD8+ T cells can recognize neoantigens on cancer cells and mediate direct cytotoxicity by releasing death-inducing granules, including granzymes and perforin (30). Additionally, CD8+ T cells secrete interferon-gamma (IFN-γ), which functions in tumor immunosurveillance and tumor immunogenicity. Moreover, sustained and effective CD8+ T cell responses are supported by CD4+ T cells via priming, indirect cytokine release, and prevention of cytotoxic T lymphocyte exhaustion (31–33).

IBC has a distinct pattern of T-cell expression and regulation (4). In flow cytometric analyses of patient blood, there are lower percentages of CD4+ and CD8+ cells that have been activated by the T cell receptor (TCR) in IBC patients compared to non-IBC patients and healthy controls, respectively (4, 34, 35). Furthermore, patients with stage IV IBC show significant decreases in all types of circulating CD4+ T-cells, as well as in memory CD8+ T-cell subsets. Lymphopenia is a key distinctive feature of immune cell profiles in metastatic IBC patients (34).

In IBC, cancer cells can exploit the PD-1/PD-L1 axis. Upregulation of PD-L1 facilitates immune evasion and creates an immunosuppressive tumor microenvironment (31, 36). Patient cohort studies have shown higher PD-L1 expression in IBC than in non-IBC (37). This increase is also positively correlated with PD-1 expression in stage IV IBC patients (34). Increased PD-L1 in IBC patients is associated with high levels of stromal tumor-infiltrating lymphocytes (sTIL), including both CD3+ and CD8+ T-cells (37, 38). It also correlates with alterations in CD8+ T-cell subsets, including shifts toward effector memory phenotypes (34). IBC tumors are enriched with CD4+ and CD8+ T-cells, which reflects sTIL levels similar to non-IBC (4, 38). Recruitment of immune cells may be partially independent of systemic or peripheral activity (34). Moreover, PD-L1-positive IBC tumors show upregulation of T-cell-specific gene markers for signaling, differentiation, and cytotoxic function in transcriptomic analyses. These include IFN-alpha, IFN-gamma, and TNF-alpha, as well as higher expression of perforin and granzyme B (37).

Meanwhile, PD-L1-expressing IBC samples also displayed overexpression of T-cell exhaustion markers, including CTLA4 and LAG3 (37). One proposed, and at present correlative, sequence is that enrichment of CD8+ effector memory T-cell subsets (hence the increase in T-cell activity markers) (34) is followed by a PD-L1-associated shift towards an exhausted state (possibly as a protective response to this influx of infiltrating T-cells) (31, 36, 37), which may ultimately account for an immunosuppressive TME, metastasis, and decline in overall T-cell numbers (4, 34). These steps have been observed as cross-sectional associations rather than demonstrated as a causal chain. Several immunotherapeutic studies have targeted the PD-1/PD-L1 interaction in IBC to reactivate previously engaged T cells and their antitumor response; the IBC-specific experience to date derives from early-phase studies, including a phase II trial of pembrolizumab reported in abstract form (5, 36). In practical terms, the therapeutic implication for IBC can be stated briefly: the coexistence of an activated effector compartment with exhaustion-marker expression identifies a plausible substrate for checkpoint blockade, but the IBC-specific clinical evidence remains confined to early-phase studies, and no checkpoint inhibitor carries an IBC-specific indication. Current use therefore follows the subtype-based approvals summarized in the treatment section below.

These activated T-cells and their cytokine production have also been implicated in the etiology of IBC via alterations in the TME. Specifically, *in vitro*, TNF-alpha, IL-6, and TGF-beta sourced from activated immune cells induce epithelial-to-mesenchymal transition (EMT) in IBC cells, which plays a key role in metastasis and stem-cell resistance to treatment (39). Interestingly, a hallmark of IBC regarding EMT induction/signaling is that the cytokines also lead to concomitant overexpression of E-cadherin, an adhesion molecule that might be responsible for the clustering phenotype characteristic of IBC tumor emboli (39, 40). Th17 cell populations have dual functions in tumor development: stimulating immune cells in the TME to become the Th1 phenotype and produce abundant IFN-γ to suppress tumor growth, while also secreting IL-17 to promote tumor angiogenesis and negatively affect immune surveillance (41, 42).

### B cells in aggressive breast cancers

Tumor-infiltrating B lymphocytes (TIL-Bs) play an important role in the TME by exerting antitumor functions through direct tumor killing, antigen presentation, antibody production, and cytokine secretion (43, 44). Analysis of biopsies from IBC patients has shown that CD20+ TIL-Bs are an independent favorable prognostic factor in IBC, suggesting a potential antitumor role for B-cells in the IBC TME, possibly by enhancing cytotoxic T-cell activity (38, 45). Furthermore, a meta-analysis of 2628 patients from eight clinical studies has shown that higher TIL-B levels were associated with favorable overall survival (OS), breast cancer-specific survival (BCSS), and disease-free survival (DFS) (46). As with T-cells, B-cell infiltration in IBC is also associated with PD-L1-expressing TILs. Notably, PD-L1 expression in IBC is also associated with increased expression of B-cell-specific gene signatures (37). Correspondingly, both PD-1 and PD-L1 expression in stage IV IBC were significantly correlated with CD20+ B-cell infiltration (34, 37).

In patient cohorts, IBC and triple-negative IBC (TN-IBC) patients who harbor both CD20+ TILs and PD-L1+ TILs show improved DFS and BCSS compared with patients lacking both, or having either one alone; importantly, this association is seen in IBC but not in locally advanced breast cancer, implicating differences in the TME between IBC and locally advanced breast cancer that are potentially linked to B-cell and PD-L1 function (4, 38). In addition, compared with non-IBC, one study found higher levels of memory B cells in IBC, a finding that was accompanied by higher PD-L1 expression (37). Together, these prognostic associations indicate that, alongside PD-L1-directed immunotherapy, the role of B-cells and their specificity and modulation is an area for further study and therapeutic development, particularly regarding B-cell activation; no B-cell-directed strategy has yet entered clinical testing in IBC.

### Tumor-associated macrophages

Tumor-associated macrophages (TAMs) are key players in the IBC TME (4). CD68 and CD163 are notably expressed by TAMs in the IBC TME (47). Preclinical studies showed that IBC-proximal TAMs mimic M2 macrophages (47), including immunosuppressive features, tumor extracellular matrix (ECM) remodeling, and maintaining vascular patency, contributing to cancer cell survival (48). Preclinical research has unveiled that TAMs can express high levels of the gene encoding the AXL ligand growth arrest-specific protein 6 (GAS6) (49). GAS6 is known to foster the proliferation and growth of various cancers, including IBC in murine and *in vitro* models (50). This work supports the direct involvement of TAMs in enhancing the survival of breast cancer cells.

In an *in vivo* IBC model, anti-colony-stimulating factor 1 (CSF-1), a factor involved in M2 macrophage polarization, reduced the aggressive clinical phenotype (51). Furthermore, *in vitro* experiments showed that IL-6 secretion from epithelial cells and mesenchymal stem cells (MSCs) can affect TAM infiltration (47, 51). Consequently, anti-IL-6 antibodies may restore treatment sensitivity, reducing the clinically aggressive phenotype of IBC, a hypothesis supported to date only by these preclinical data (51). These findings supported the clinical-trial testing of anti-TAM strategies along with immune checkpoint inhibitors, including the AXL inhibitor bemcentinib with pembrolizumab in TNBC and triple-negative IBC (phase II, NCT03184558) and TME-directed combinations in the Morpheus-panBC umbrella study (NCT03424005); these registry entries document trial conduct, not efficacy (52, 53).

### Tumor-associated neutrophils

Historically, tumor-associated neutrophils (TANs) were considered bystanders. However, recent evidence suggests that TANs are associated with unfavorable outcomes in solid tumors, largely from computational deconvolution of bulk transcriptomes across cancer types (54). Just like TAMs, TANs undergo polarization into the pro-tumorigenic N2 phenotype, as characterized in murine models (55). N2 TANs promote immunosuppression, tumor growth, angiogenesis, and metastasis by inducing DNA instability and releasing immunosuppressive cytokines and chemokines (56).

Analysis of 105 patients with localized breast cancer (stage I-III) showed that the percentage of TAN positivity was highest in triple-negative breast cancer (88%), followed by HER2+ breast cancer (53%). Interestingly, only 5% of hormone receptor-positive (HR+) breast cancer patients were TAN-positive, whereas HR negativity was associated with significantly higher TAN positivity (57). To date, these studies are not IBC-specific, and more studies are required to understand the detailed role of TANs in IBC biology; any therapeutic relevance of TANs in IBC is therefore extrapolated from non-IBC breast cancer populations rather than demonstrated in the disease itself.

The intricacies of neutrophil-dependent carcinogenesis are context-dependent. For instance, in murine models, while CXCR2 aids neutrophil migration to cancer-promoting sites, its knockdown in neutrophils enhances reactive oxygen species (ROS) production and thus has a cancer-promoting effect (58–60). Genetically engineered mouse models (GEMMs) may provide an exploration of neutrophil biology related to cancer by manipulating neutrophils and the factors they produce as cancer spontaneously develops, thereby providing a robust platform for future studies (61).

### Stromal, vascular, and spatial dimensions of the IBC microenvironment

Immune cells are only part of the IBC ecosystem. Cancer-associated fibroblasts and mesenchymal stromal cells participate in the chemokine and IL-6 networks that polarize macrophages and support the mesenchymal state of IBC cells (47, 51), and endothelial and lymphatic vessels are central to the defining lesion of the disease: lymphovascular tumor emboli, whose formation, vascular invasion, and dependence on macrophage interactions have been modeled in microfluidic and preclinical systems (48). Extracellular matrix remodeling and hypoxia within the dermal lymphatic niche further condition therapeutic access and immune exclusion, and the E-cadherin-retaining, clustered phenotype of emboli ties the stromal compartment directly to the EMT biology described above (39, 40). Finally, immune activity in IBC is spatially heterogeneous: infiltrate composition and checkpoint expression differ between the tumor core, the stromal interface, and the emboli themselves, which cautions against bulk metrics and motivates spatially resolved profiling as these platforms mature (4). These stromal and spatial dimensions remain largely at the preclinical and discovery stage, but any account of therapeutic resistance in IBC that omits them is incomplete.

## Emerging therapeutic directions

### Current subtype-directed standards as the reference point

Emerging strategies must be read against what is already established. In advanced disease, current standards are subtype-directed: endocrine therapy with CDK4/6 inhibition in HR-positive/HER2-negative disease, with elacestrant available for ESR1-mutant tumors and alpelisib or capivasertib for PIK3CA/AKT pathway alterations; dual HER2 blockade and HER2-directed antibody-drug conjugates (ADCs) in HER2-positive disease; pembrolizumab with chemotherapy in PD-L1-positive metastatic TNBC and in high-risk early TNBC; and PARP inhibitors for germline BRCA1/2 carriers (62). IBC itself has no subtype-specific approved therapy: standard management is trimodality treatment, neoadjuvant systemic therapy according to receptor subtype, modified radical mastectomy, and comprehensive locoregional radiotherapy (63). **Table 1** summarizes the regulatory and developmental status of the therapeutic strategies discussed in this review. Clinical trial registry entries are cited to document that a strategy is under clinical evaluation and are not presented as efficacy evidence.

**Table 1:** Regulatory and developmental status of therapeutic strategies discussed in this review.

Status tier	Strategies discussed in this review	Notes
Approved/guideline-endorsed	Endocrine therapy + CDK4/6 inhibitors (HR+/HER2-); elacestrant (ESR1-mutant); alpelisib, capivasertib (PIK3CA/AKT pathway); dual HER2 blockade; pembrolizumab (PD-L1+ mTNBC; high-risk early TNBC); PARP inhibitors (germline BRCA1/2); ADCs: T-DM1, T-DXd, sacituzumab govitecan, datopotamab deruxtecan (indication-specific)	Established in defined populations per current guidelines; none is IBC-specific (62). Atezolizumab: US accelerated approval in mTNBC withdrawn.
Clinical-trial strategies	Trastuzumab deruxtecan plus durvalumab in stage III HER2-expressing IBC (TRUDI, phase II, NCT05795101); pembrolizumab in metastatic IBC (phase II, abstract) (5); bemcentinib (AXL inhibition) plus pembrolizumab in TNBC/TN-IBC (phase II, NCT03184558) (52); sacituzumab govitecan (NeoSTAR, NCT04230109) and panitumumab (NCT05177796) with neoadjuvant immunotherapy in IBC; TME-directed combinations, Morpheus-panBC (NCT03424005) (53); ctDNA-guided treatment escalation at molecular relapse (multiple ongoing trials) (13, 14)	Under prospective evaluation; registry entries and abstracts document trial conduct, not efficacy; not for routine use. TRUDI is the only IBC-specific checkpoint inhibitor combination currently accruing
Preclinical concepts	Anti-IL-6/anti-CSF-1 macrophage-directed strategies (47, 51); CXCR2/neutrophil modulation (58–60); miRNA replacement or inhibition and other RNA therapeutics (64–75)	Mechanistic and animal-model evidence only; delivery, specificity, and reproducibility barriers unresolved

ADC, antibody-drug conjugate; mTNBC, metastatic triple-negative breast cancer; TN-IBC, triple-negative inflammatory breast cancer; superscript numerals refer to the reference list.

### New diagnostics and therapeutics in RNA

MicroRNAs (miRNAs) mediate the degradation and silencing of messenger RNAs, with (64, 65) downstream effects on proliferation, apoptosis, and differentiation, and more than 1000 human miRNAs identified to date (64–66). Foundational profiling studies established aberrant miRNA expression (including miR-125b, miR-145, miR-155, and miR-21) in breast cancer relative to normal tissue, with expression profiles predicting receptor status, stage, and proliferative index, and miR-21 in particular carrying prognostic associations (67–69). Preclinical work subsequently demonstrated oncogenic and tumor-suppressive miRNA functions and showed that artificial miRNAs, antisense inhibition, and liposomal or viral delivery can reduce invasion, metastasis, and chemoresistance in cell lines and murine models (70–75).

It must be emphasized that essentially all miRNA replacement and inhibition strategies in breast cancer remain preclinical, and the field’s foundational profiling literature dates largely from 2005 to 2015. The barriers to clinical translation are well defined: delivery to tumor tissue, nuclease stability, off-target and immunostimulatory effects, tumor specificity, and reproducibility across models. The contemporary RNA field has meanwhile broadened beyond miRNAs to circulating and extracellular-vesicle-associated RNAs as candidate liquid-biopsy analytes, long non-coding RNAs as regulators and biomarkers, and chemically modified antisense oligonucleotides and siRNA with improved delivery chemistry; in breast cancer, these too remain investigational, and no RNA therapeutic is approved in this disease. Given this maturity gap, RNA-based approaches are best regarded as a discovery pipeline feeding the diagnostic and therapeutic strategies discussed above, rather than as near-term clinical options.

### Integration of ADC treatment in advanced breast cancer

As the therapeutic landscape of breast cancer is undergoing rapid transformation, ADCs represent a particularly promising class of therapeutics. ADCs combine a monoclonal antibody targeting tumor-associated antigens with a potent cytotoxic payload, enabling selective delivery of chemotherapy to malignant cells while also exerting a bystander effect on adjacent tumor cells (76). This targeted approach has shown substantial efficacy across multiple biologically defined breast cancer subtypes; however, data supporting its use in aggressive phenotypes such as IBC remain limited, since IBC patients are under-represented in the registration trials and ADC efficacy in IBC has not been separately established.

Currently, four ADCs have received regulatory approval for the treatment of breast cancer. Ado-trastuzumab emtansine (T-DM1) is the only ADC approved for early-stage HER2+ breast cancer (76). Trastuzumab deruxtecan (T-DXd), also directed against HER2, has redefined standards of care for patients with HER2-positive disease in the metastatic setting (77). The DESTINY-Breast clinical trial program has consistently demonstrated its superiority over T-DM1, with substantial improvements in progression-free and overall survival (77). In the DESTINY-Breast04 trial, T-DXd showed efficacy in patients with HER2-low disease as well. Sacituzumab govitecan (SG), a TROP2-directed ADC with an SN-38 payload, was the first ADC approved for metastatic triple-negative breast cancer (mTNBC) based on the ASCENT trial and has since expanded its indications to include HR+/HER2- disease after multiple prior lines of therapy (77). Datopotamab deruxtecan (Dato-DXd), another TROP2-targeting ADC but with a deruxtecan payload, has shown significant improvements in progression-free survival compared with physician’s choice chemotherapy in patients with HR+/HER2- disease following endocrine therapy and prior chemotherapy (77). Collectively, these agents provide new therapeutic opportunities for populations that historically have had limited options; their evaluation in IBC-enriched cohorts is a priority for the field.

## Conclusions

Managing IBC and other advanced breast cancers from diagnosis through treatment is complex, and the evidence supporting each proposed solution stands at a different stage of maturity. On the diagnostic side, ctDNA-based monitoring has demonstrated prognostic validity, and the first evidence of clinical utility has emerged for ctDNA-guided endocrine switching; standardized intervention thresholds, validated assays for low-shedding disease, and interception trials in rapidly progressing subtypes are the outstanding needs, and the narrow interception window of IBC makes it both the hardest and the most informative setting in which to close them. On the biological side, the IBC TME literature, spanning T cells, B cells, macrophages, neutrophils, and the stromal and vascular compartments, is rich in prognostic associations but thin in causal mechanism and thinner still in therapies: no TME-directed strategy has advanced beyond early-phase trials in this disease. The principal evidence gaps are structural and worth naming: the rarity and heterogeneity of IBC, small and often single-institution cohorts, limited prospective validation, the absence of validated IBC-specific biomarkers, and the translational gap between immune profiling and effective treatment. What is established now is subtype-directed systemic therapy, trimodality management of IBC, and the approved ADCs; what constitutes the research agenda is molecular interception, TME-directed combinations, and RNA-based approaches. Keeping that boundary explicit, as this review has attempted to do, is itself a contribution to a field in which the distance between promise and practice is easily understated.
